# *Aedes albopictus* microbiome derives from environmental sources and partitions across distinct host tissues

**DOI:** 10.1002/mbo3.1364

**Published:** 2023-06-12

**Authors:** Priscilla S. Seabourn, Danya E. Weber, Helen Spafford, Matthew C. I. Medeiros

**Affiliations:** ^1^ Plant and Environmental Protection Sciences Honolulu Hawaii USA; ^2^ Pacific Biosciences Research Center University of Hawaii Honolulu Hawaii USA; ^3^ Department of Primary Industries and Regional Development South Perth Western Australia Australia; ^4^ Center for Microbiome Analysis through Island Knowledge and Investigation University of Hawaii at Manoa Honolulu Hawaii USA

**Keywords:** *Aedes albopictus*, environment, microbiome assembly, tissue microbiome

## Abstract

The mosquito microbiome consists of a consortium of interacting microorganisms that reside on and within culicid hosts. Mosquitoes acquire most of their microbial diversity from the environment over their life cycle. Once present within the mosquito host, the microbes colonize distinct tissues, and these symbiotic relationships are maintained by immune‐related mechanisms, environmental filtering, and trait selection. The processes that govern how environmental microbes assemble across the tissues within mosquitoes remain poorly resolved. We use ecological network analyses to examine how environmental bacteria assemble to form bacteriomes among *Aedes albopictus* host tissues. Mosquitoes, water, soil, and plant nectar were collected from 20 sites in the Mānoa Valley, Oahu. DNA was extracted and associated bacteriomes were inventoried using Earth Microbiome Project protocols. We find that the bacteriomes of *A. albopictus* tissues were compositional taxonomic subsets of environmental bacteriomes and suggest that the environmental microbiome serves as a source pool that supports mosquito microbiome diversity. Within the mosquito, the microbiomes of the crop, midgut, Malpighian tubules, and ovaries differed in composition. This microbial diversity partitioned among host tissues formed two specialized modules: one in the crop and midgut, and another in the Malpighian tubules and ovaries. The specialized modules may form based on microbe niche preferences and/or selection of mosquito tissues for specific microbes that aid unique biological functions of the tissue types. A strong niche‐driven assembly of tissue‐specific microbiotas from the environmental species pool suggests that each tissue has specialized associations with microbes, which derive from host‐mediated microbe selection.

## Graphical abstract

The processes that govern how environmental microbes assemble across the tissues within mosquitoes remain poorly resolved. We use ecological network analyses to examine how environmental bacteria assemble to form bacteriomes among *Aedes albopictus* host tissues. Broadly, these data demonstrate that mosquito tissue microbiomes are nested within environmental sources, and form specialized modules based on tissue type.

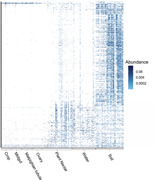

## 1 INTRODUCTION

Host‐associated microbiomes or symbionts (i.e., organisms that rely on organisms as a habitat) are diverse ecological communities comprised of fungi, viruses, archaea, bacteria, and protozoans that often provide essential functions to their metazoan hosts (Douglas, 2015; Overstreet & Lotz, 2016). For instance, microbiome diversity has been attributed to many phenotypes that underlie overall host function, including nutrient provisioning (Gupta & Nair, 2020), metabolism (Wilke & Marrelli, 2015), and protection from natural enemies (Hegde et al., 2015; Hill et al., 2014; Tol & Dimopoulos, 2016). A primary feature of these host‐associated microbiomes is substantial ecological diversity, including a large number of symbiont taxa in a microbiome assemblage and high turnover in the composition of these taxa among individual host microbiome assemblages (Alfano et al., 2019; Gupta & Nair, 2020). The distribution and assemblage of microbes change in hosts through space and time, and understanding the drivers of community assembly remains one of the significant challenges in microbiome community ecology and has important implications for disease‐mitigating strategies such as paratransgenesis (Gao et al., 2021; Jayakrishnan et al., 2018; Saab et al., 2020; Wilke & Marrelli, 2015). This is especially the case for complex organisms such as mosquitoes (Diptera: Culicidae) that spend time in both terrestrial and aquatic environments, metamorphose during their life cycle, and require discrete sources of food for maintenance and reproduction. Given this complexity, it is expected that the microbiota and their functional value in mosquitoes will change developmentally and even spatially, within the host.

The microbiome of mosquitoes consists of diverse populations of interacting symbiotic microorganisms that reside in mosquito hosts and may distribute differentially across mosquito tissues. The mosquito microbiome is a major modulator of host nutrition, metabolism, reproduction, development, immunocompetence, and behavior (Wang et al., 2011; Weiss & Aksoy, 2011). These effects can scale to impact the capacity of mosquito vectors to transmit infectious diseases (Alfano et al., 2019). Previous studies have demonstrated high diversity in the structure and composition of mosquito‐associated microbial communities among mosquito species (Guégan et al., 2018; Wang et al., 2011), geographic sites (Muturi et al., 2017; Schrieke et al., 2022), developmental life stages (Wang et al., 2011), host sex (Wang et al., 2011), and host tissue (Minard et al., 2018). Except for endosymbiotic *Wolbachia* sp., which is acquired early on in embryogenesis, mosquitoes likely acquire the majority of this diversity from their environment (Duguma et al., 2013; Mancini et al., 2018). Indeed, recent studies have highlighted mosquito microbiota demonstrating habitat specificity (Schrieke et al., 2022). However, the environmental sources of these facultative symbionts and how they assemble within mosquito hosts remain inadequately resolved (Tawidian et al., 2021).

Mosquitoes interact with different microbial‐rich environmental substrates at different stages of their amphibious life cycle. Research has shown that mosquito larvae have similar microbiome composition to their aquatic habitats (Dennison et al., 2014). During the terrestrial adult life stage, a portion of the gut bacteria of mosquitoes, such as *Asaia* sp., are acquired from the microbiomes that form on sugary plant nectars (Bassene et al., 2020). Indeed, removing mosquitoes from natural settings and allowing their microbiome to recruit in more sterile lab settings is associated with dramatic shifts in the mosquito microbiome (Akorli et al., 2019; Saab et al., 2020). Broadly, these examples suggest that the environment plays a major role in structuring mosquito microbiotas and may be the ultimate source of its diversity (Tawidian et al., 2021; Thongsripong et al., 2018).

As microorganisms from the environment colonize mosquitoes, they may sort differentially among organs and tissue types. For example, a recent study found crop and midgut‐specific microbiotas within *Aedes* mosquitoes, which may be due to mosquito physiology (Villegas et al., 2023). The mechanisms and consequences of this assembly process remain inadequately described in mosquitoes. However, research in other invertebrate systems indicates that such differentiation might be related to niche effects associated with the microbe in different environments within the host as well as host‐mediated microbe selection during the formation of the symbiosis. For example, *Euprymna scolopes* (Sepiida: Sepiolidae) is exposed to millions of bacteria in seawater, yet only a single strain of *Vibrio fisheri* (Vibrionales: Vibrionaceae) colonizes the light organ (Mandel & Dunn, 2016; Ruby & Lee, 1998). Previous studies have demonstrated that the host sets up a series of chemical and physical barriers to microbial colonization that selectively promote the assembly of *V. fisheri* and inhibit the colonization of potential microbial competitors in the light‐organ environment. Within the light organ, *V. fisheri* bioluminesces and reduces host mortality associated with predation through countershading in the water column. This system indicates that invertebrate hosts may actively develop and maintain symbiosis in distinct host tissues by selecting microbes that provide a beneficial function (Brooks et al., 2016).

Here, we use ecological network analyses (including nestedness, modularity, and specialization) to characterize attributes of the *Aedes albopictus* microbiome among individual host tissues and the mosquito's environment to better understand how mosquito‐associated symbionts source from environmental substrates and partition among different host tissues (Amend et al., 2019; Dormann & Strauss, 2014; Wright et al., 1997). Our study estimates nestedness, modularity, and specialization within the *A. albopictus* mosquito as a model. This mosquito is widespread globally, a medically relevant vector of several arboviruses, found across diverse habitat types, and previous studies have shown high diversity in the microbiome among individual hosts within and among populations (Medeiros et al., 2022; Seabourn et al., 2020). Overall, our analyses test a series of hypotheses that purport how microbes source and disseminate from the environment across distinct tissues of the mosquito host. We hypothesize that the environment is a source of microbes that enter a symbiosis with mosquitoes, and we predict that the lower diversity mosquito microbiomes will be a nested taxonomic subset of the higher diversity environmental microbiomes. We further hypothesize that these microbes will partition differentially within the mosquito among different organs and tissues and that a pattern of differential assortment among tissues may suggest different underlying ecological mechanisms of assembly. For instance, nested microbiomes along the mosquito alimentary canal may form in response to strong forces of dispersal from environmental sources and host tissue‐specific filtering in the distinct tissues (Amend et al., 2019). Alternatively, very strong patterns of tissue‐specific filtering would overwhelm the role of dispersal and lead to specialization and/or modularity among mosquito tissue microbiomes, perhaps associated with the biological functions of each tissue. Clarifying aspects of the environment that contribute to mosquito microbiomes will shed light on the origin of microbial symbionts and their process of assembly to form a host‐associated microbiome and can be used to inform vector control strategies such as paratransgenesis (Gao et al., 2021; Wang et al., 2017; Wilke & Marrelli, 2015).

## 2 METHODS

### 2.1 Mosquito and environmental sampling

Adult *A. albopictus* mosquitoes and environmental samples were collected at 20 sites within two main areas associated with the University of Hawai'i at Mānoa (main campus and Lyon Arboretum) in Mānoa Valley on Oahu over 6 months in 2019. Sampling sites were spaced at least 30 m apart from each other. The sites were selected to represent typical urban to natural environments present in Hawaii with known *A. albopictus* mosquito presence (Supporting Information: Table S1). Sites 1–9, 15, and 20 were primarily urban and were proximal to human habitation with natural and artificial mosquito oviposition sites detected (Supporting Information: Table S1). Sites 10–14 and 16–19 were in a natural green space that had varied levels of human visitation and use. Sites 10–12 were located along a frequently visited trail, while sites 13, 14, and 16–19 were approximately 30 m from the trailhead with dense vegetation and reduced likelihood of encounters with people. Each site was sampled once between June 2019 and December 2019 (Supporting Information: Table S1).

At each site, mosquitoes were collected using a battery‐powered aspirator at one time point at each site (Supporting Information: Table S1). Samples were immediately placed in a plastic vial and placed in an insulated cooler with ice. The mosquitoes were identified as species using the Darsie and Ward taxonomic key (Darsie, 2005), and their sex and gravid status was determined using a standard ×10–40 dissecting scope. Female *A. albopictus* that were fed and/or gravid (determined by the presence of an enlarged abdomen) were excluded from downstream processing.

From the sample, a total of five host‐seeking female mosquitoes were selected for further analysis. The samples were kept on ice and were processed within 4 h of collection. Individual mosquitoes were dissected to extract tissues for genetic analysis. Before dissection, whole mosquitoes were surface sterilized with a 75% ethanol wash, followed by two rinses in sterile 1× phosphate‐buffered saline. Each mosquito was placed on ice and dissected with EMS High Precision and Ultra Fine Tweezers (Electron Microscopy Sciences) to expose the internal organs for dissection. The crop, midgut, Malpighian tubules, and ovaries were removed and placed separately in a sterile 1.5 microcentrifuge tube in the −80°C freezer for storage (i.e., a single tube for each tissue section). Samples of the same tissues from five same individual females captured at the same sampling event were pooled (i.e., crop, midgut, Malpighian tubule, and ovary tissue pools from the same five females collected from the same site), placed in a sterile 1.5 microcentrifuge tube, and stored at −80°C. For each of the 20 sites, there was a single sample for each of the four tissue types. However, the ovarian tissue sample from Site 1 failed to exit the molecular pipeline described, leaving a total of 79 mosquito tissue samples for analysis.

At each sampling event, samples were collected from environmental substrates that mosquitoes interact with during their life cycle, including plant nectar (a common food source shared by both sexes), soil (common resting site and constituent of larval habitat), and standing water (major fluid medium of the larval habitat). These samples provided sources of environmental bacteria from which DNA could be extracted. Where possible, environmental samples were collected in triplicates (i.e., three soil samples per site, three plant nectar samples from each site, three water samples per site where possible) on the same day and within 8–10 m of mosquito captures. A total of 159 environmental samples were collected throughout the study (*N* = 54 soil, *N* = 59 plant nectar, water). Approximately 250 mg of soil was collected at the surface of the ground at each site with a clean metal spoon (i.e., surface sterilized with 70% ethanol before collection) and placed in a sterile 1.5 mL microcentrifuge tube. Substrates that produce plant nectar, herein referred to as “plant nectar sources” (abbreviated as PNS), were collected by rubbing sterile flocked swabs (Puritan) on flower nectaries and the exudate of fallen fruits (Supporting Information: Table S2). Flocked swabs were rubbed against the surface of a single flower or fruit 10 times while rotating clockwise and placed in a sterile microcentrifuge tube. The majority of PNS samples were collected from flowers (*n* = 55) as compared to fruit (*n* = 5). Flowers and fruit were occasionally sampled from the same tree. Where possible, 250 µL of water samples were sampled from a single larval habitat (i.e., tree holes, pooled water generally <500 mL) using sterile pipettes. All environmental samples were immediately placed in an insulated cooler with ice and transported to the laboratory and stored in a −80°C freezer. While seasonal variation likely exists, our balanced design controlled for this intrinsically (i.e., we sampled all tissues and environments at each site/time).

### 2.2 DNA extraction and sequencing

DNA was extracted using a Qiagen MagAttract PowerSoil DNA Kingfisher Kit (Qiagen) on a KingFisher Flex (Thermo Fisher Scientific) following the manufacturer's protocol. Library preparation was done using a modified version of the Earth Microbiome Project 16S V4 Illumina Amplicon Protocol. Standard Earth Microbiome Project barcoded 16S primers were used, based on 515F (Parada)‐(5′‐GTGYCAGCMGCCGCGGTAA‐3′) and 806R‐(Apprill) (5′‐GGACTACNVGGGTWTCTAAT‐3′) primers. Each PCR reaction contained the following components: 16.25 µL of nuclease‐free water, 5.0 µL of 5× KAPA HiFi Fidelity Buffer, 1.0 µL of template DNA (approximately 10–87 ng), 0.75 µL of 10 mM KAPA dNTP Mix, 0.75 µL of 10 µM forward primer, 0.75 µL of 10 µM reverse primer, and 0.5 µL of 1 U/µL KAPA HiFi HotStart DNA Polymerase. A no‐template PCR reaction was also performed as a negative reagent blank. In addition, a ZymoBIOMICS Microbial Community DNA standard was used to assess overall performance (Zymo Research). PCR amplifications were performed in an Applied Biosystems SimpliAmp Thermal Cycler (Thermo Fisher Scientific) using the following conditions: initial denaturation at 95°C for 3 min; 35 cycles of denaturation at 98°C for 20 s, annealing at 60°C for 15 s, extension at 72°C for 30 s; and a final extension at 72°C for 30 s. The PCR products were visualized on a 2% agarose gel before being purified and normalized to 1.25–2.50 ng/μL using a Just‐a‐Plate kit (Charm Biotech). The purity and concentration of a subset of samples were assessed with a NanoDrop (Thermo Fisher Scientific). A volume of 10 µL from each sample was pooled and then purified using a 1.2× volume of homemade AMPureXP Beads (Rohland & Reich, 2012). The library was checked for quality and quantity using a Bioanalyzer High Sensitivity chip (Agilent Technologies) and sequenced by Advanced Studies in Genomics, Proteomics, and Bioinformatics at the University of Hawai'i at Mānoa using Illumina MiSeq PE300 with a MiSeq Reagent Kit v3 (Illumina).

### 2.3 Bioinformatics

Bioinformatic processing of sequencing reads was performed using the bioinformatic pipeline to identify amplicon sequence variants (ASVs) (Arisdakessian et al., 2020). After quality control, sequencing resulted in 17.1 million reads (per sample mean: 59,749; per sample median: 60,128). ASVs found in the reagent negative controls and no‐template PCR controls were removed from the analysis as they represent contaminants along the molecular analysis pipeline. Exceedingly rare ASV taxa were removed if not seen more than one time (i.e., singletons), and samples found in less than 5% across all observations (i.e., rows in the data set) using the “filter_taxa” function implemented in package Phyloseq in R. Following this filter step, 153 ASVs remained in the inventoried microbiomes of mosquito tissue samples and 1055 ASVs remained among the environmental samples were used to assess diversity measurements (Supporting Information: Tables S3 and S4).

### 2.4 Statistical analysis

Nestedness describes the degree to which ecological communities with low species richness are subsets of those with higher species richness (Amend et al., 2019; Atmar & Patterson, 1993; Patterson & Atmar, 2008). A nestedness temperature (Patterson & Atmar, 2008) was estimated to test the hypothesis that low‐diversity mosquito tissue (crop, midgut, Malpighian tubule, and ovary) microbiomes source from higher diversity environmental (soil, water, and plant nectar) microbiomes. Three weighted bipartite matrices were constructed: (1) environment samples unmerged (water, soil, plant nectar) + partitioned mosquito samples (i.e., crop, midgut, Malpighian tubule, and ovary), (2) environmental samples merged + partitioned mosquito samples, (3) partitioned mosquito samples completely excluding all environmental samples. Endosymbionts of *Wolbachia* (wAlbA and wAlbB) were excluded from the nestedness analysis as the focus of the analysis was to assess if environmentally acquired symbionts are a source for mosquito microbiomes (Ding et al., 2020). To account for disparities in ASV abundances between mosquito and environmental samples, the bipartite matrices were resampled without replacement using “rarefy_even_depth” function in the phyloseq package in program R using “sample.size” of 2176 for the environment + mosquito matrix and 2000 for the mosquito matrix. The nestedness ranged from 0 (perfectly nested) to 100 (random) based on a weighted bipartite matrix, implemented in the “nestedtemp” function in the vegan package in program R (Dixon, 2003). The nested temperature was compared with a distribution of randomized null communities simulated using the “vaznull” method. The Simpson's index of each mosquito and environmental microbiota was estimated with the function “diversity” in the package vegan. Richness was measured as the total number of ASVs in a sample and was assessed on the bipartite matrices that were resampled without replacement to control for uneven sampling depth.

The QuanBiMo algorithm, following the method and code described in Dormann et al. (2009), was used to calculate modularity (*Q*) for the weighted mosquito tissue network. Modularity describes patterns that consist of partitions or modules of community members that have either (i) few to no interactions between other modules present or (ii) more interactions within an individual module (Dormann & Strauss, 2014; Patterson & Atmar, 2008). The null model function (100 randomizations; method “vaznull”) was used to convert *Q* to a *z*‐score and estimate a *p* value assuming one degree of freedom. A generalized linear mixed model (GLMM) implemented in the package *glmmTMB* in program R was used to test for the overall differences in the composition of the microbiota from mosquitoes across the four tissue types. The model included a random interaction between the tissue type variable and symbiont taxa, which permit the different symbiont taxa to have different responses in relative abundance between the different tissues and a zero‐inflation component, which was allowed to vary across the levels of the symbiont taxa.

GLMMs were also used to understand how variance in the composition of the tissue‐specific mosquito microbiomes was partitioned among hypothetical groupings of tissues. We created 14 dummy variables that included all potential groupings of the four tissue types, as well as a null model that included no tissue grouping variable and only a y‐intercept (Table 1).

**Table 1:** Fourteen models with hypothetical groupings of tissues.

Model	Grouping
cg‐mo	Two modules: crop + midgut; Malpighian tubules + ovaries
c‐g‐mo	Three modules: crop; midgut; Malpighian tubules + ovaries
m‐o‐cg	Three modules: Malpighian tubules; ovaries; crop + midgut
c‐g‐m‐o	Four modules: crop; midgut; Malpighian tubules; ovaries
g‐o‐cm	Three modules: midgut; ovaries; crop + Malpighian tubules
c‐o‐gm	Three modules: crop; ovaries; midgut + Malpighian tubules
o‐cgm	Two modules: ovaries; crop + midgut + Malpighian tubules
c‐m‐go	Three modules: crop; Malpighian tubules; midgut + ovaries
g‐m‐co	Three modules: midgut; Malpighian tubules; crop + ovaries
g‐cmo	Two modules: midgut; crop + Malpighian tubules + ovaries
m‐cgo	Two modules: Malpighian tubules; crop + midgut + ovaries
c‐gmo	Two modules: crop; midgut + Malpighian tubules + ovaries
cm‐go	Two modules: crop + Malpighian tubules; midgut + ovaries
co‐gm	Two modules: crop + ovaries; midgut + Malpighian tubules

Abbreviations: c, crop; g, midgut; m, Malpighian tubule; o, ovary.

All models except the null model include a random interaction between the tissue grouping variable and symbiont taxa, which permits each symbiont taxa to have different responses in relative abundance between the tissue groups. Every model, including the null model, incorporated a fixed intercept, a fixed effect for the tissue grouping variable, a random intercept for symbiont taxa, and a zero‐inflation component, which was allowed to vary across the levels of the symbiont taxa. Candidate models with different tissue grouping schemes were compared based on model fit, using corrected Akaike information criterion (AICc), and implemented in the package *bbmle*. The model with the lowest AIC value was the best‐fitted model. The fits of all models within 2 ΔAIC units were considered indistinguishable. ΔAICc > 2 were considered to have a better fit. Models with ΔAICc > 7 were considered a poor fit and rejected. Nonmetric multidimensional scaling (NMDS) analysis using the Bray–Curtis dissimilarity matrix was applied to visualize the similarity of tissue‐specific mosquito microbiomes using the package “vegan” in program R.

Specialization is a metric that evaluates the extent to which microbial taxa are exclusively associated with a particular category of the host (Dormann et al., 2009). The extent to which certain tissues select for microbes was calculated using the network‐wide *H*2 index under the “H2fun” in package vegan (Blüthgen et al., 2006). The *H*2 index returns a value from 0 (generalized) to 1 (specialized). Deviations from null expectations were quantified using a distribution of null community matrices calculated using the quantitative r2table method in package vegan (Amend et al., 2019). The significance of individual independent effects on α‐diversity indices was assessed through a log‐likelihood ratio test that compared a full model and a nested model that lacked an independent variable of interest. We assumed the log‐likelihood approximates using a *χ* ^2^ distribution. The conditional modes from these models were used to estimate the responses of each symbiont to each tissue type.

## 3 RESULTS

### 3.1 Mosquito and environmental microbiome

**Figure 1:**
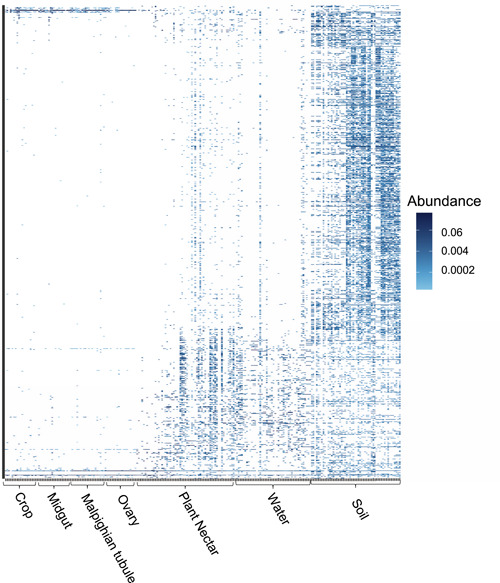
Heatmap showing the prevalence of bacterial taxa within environmental samples (soil, plant nectar, and water) and mosquito tissues (crop, midgut, Malpighian tubules, and ovaries). Cell values are calculated as proportions across rows.

A total of 16,495 bacterial ASVs (Supporting Information: Table S3, 15,855 from the environmental samples; 640 from the mosquito tissues) were identified from the pooled tissues of 79 *A. albopictus* mosquito crop (*N* = 20), midgut (*N* = 20), Malpighian tubules (*N* = 20), ovaries (*N* = 19), and 159 environmental samples (soil, plant nectar, and water). A total of 1053 bacterial ASVs were assessed and included 20 phyla (Acidobacteria, Actinobacteria, Bacteroidetes, BRC1, Chloroflexi, Cyanobacteria, Deinococcus‐Thermus, Elusimicrobia, Entotheonellaeota, Firmicutes, Fusobacteria, Gemmatimonadetes, Latescibacteria, Nitrospirae, Planctomycetes, Proteobacteria, Rokubacteria, Tenericutes, Thaumarchaeota, and Verrucomicrobia) and one unclassified bacteria phylum. Of the bacterial ASVs from the resampled (i.e., “rarefied”) data set, 192 families were identified (Figure 1). Of the ASVs from the resampled data set, the number of predominant ASVs by sample type is as follows: 1006 soil ASVs; 719 PNS ASVs; 635 water ASVs; and 153 mosquito tissue ASVs. ASV co‐occurrence was noted between environmental and mosquito sample types (Tables 2 and 3). For instance, there was higher sharing between the crop and the environmental substrates compared to the midgut, Malpighian tubule, and ovary (Table 2). In contrast, the ovary had the least amount of ASV co‐occurrence with environmental substrates (Table 2). Similar patterns of co‐occurrence were noted between the midgut and Malpighian tubules and the environment. The environmental ASVs from the resampled data set had an average (±SEM) species richness of 6.1 ± 0.01 and evenness (Simpson's index) of 0.99 ± 0.001. The resampled mosquito ASVs had an average species richness of 1.24 ± 0.006 and evenness (Simpson's index) of 0.77 ± 0.002. The crop had the highest diversity (*n* = 83 bacterial symbionts), followed by the Malpighian tubules (*n* = 63), midgut (*n* = 59), and ovaries (*n* = 38).

**Table 2:** ASV co‐occurrence of environmentally acquired symbionts among mosquito tissue types.

	Crop (*N* = 83)	Midgut (*N* = 59)	Malpighian tubule (*N* = 63)	Ovary (*N* = 38)
Plant nectar	71 (0.85)	50 (0.85)	48 (0.76)	30 (0.79)
Water	57 (0.69)	40 (0.68)	42 (0.67)	27 (0.71)
Soil	72 (0.86)	44 (0.75)	53 (0.84)	29 (0.76)

*Note*: The number within parentheses below each tissue is the total number of ASVs in each tissue. Numbers within parenthesis within the cells indicate the proportion of the tissue‐specific microbiome that is shared with the environmental microbiome. Vertically transmitted symbionts wAlbA and wAlbB were removed to curate this table. Data in the table corresponds to full data sets that have not been resampled.

Abbreviation: ASV, amplicon sequence variant.

**Table 3:** Number of ASVs that co‐occur among environmental sample types (plant nectar, water, and soil).

	Plant nectar	Water
Plant nectar (*N* = 719)		
Water (*N* = 635)	478 (0.75)	
Soil (*N* = 1006)	675 (0.67)	591 (0.88)

*Note*: The number within parentheses below each environmental sample type is the total number of ASVs in each sample.

Abbeviation: ASV, amplicon sequence variant.

### 3.2 Mosquito microbiome

**Figure A1:**
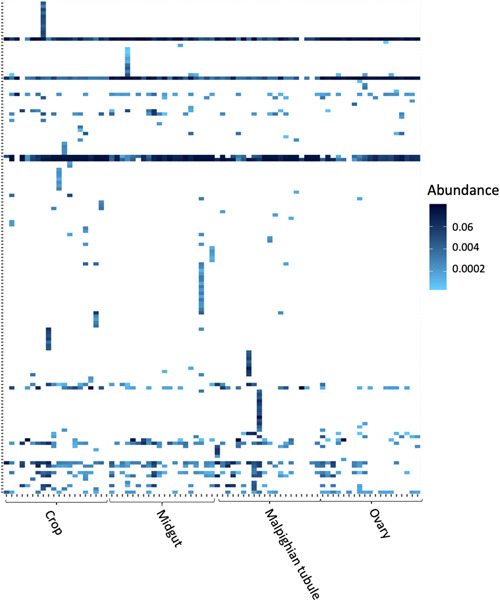
Heatmap showing the prevalence of bacterial taxa distributed across *Anopheles albopictus* tissues (crop, midgut, Malpighian tubules, and ovaries). Cell values are calculated as proportions across rows.

A GLMM used to test the variation in the composition of the microbiome among mosquito tissues (i.e., crop, midgut, etc.) indicated significant variation across tissue types (*p* < 0.0001; Figure A1 and Supporting Information: Table S5). The conditional modes of the most likely model indicate varied responses of bacterial symbionts to each tissue type (Supporting Information: Table S5). For instance, the conditional modes for the random interaction effect of bacterial symbionts and tissue type indicated that *Asaia* sp., ERWIN1 (an unclassified Erwiniaceae lineage), *Mesorhizobium* sp., ENTERO 1 (an unclassified Enterobacteriaceae lineage), HALO1 (an unclassified Halomonadaceae lineage), and *Bradyrhizobium* sp. were more abundant in the crop relative to the population average across all tissue types (i.e. crop, midgut, Malpighian tubule, and ovaries). Similarly, the interaction effect of bacterial symbiont and tissue type indicated a higher abundance of a *Cloacibacterium* sp., *Asaia* sp., and ACIDO1 (an unclassified Acidobacteria lineage) in the midgut relative to the population‐level average. The Malpighian tubules were associated with a greater presence of *Wolbachia* sp. wAlbB, *Xanthobacter* sp., and *Sphingomonas* sp., and similar to the Malpighian tubules, the ovaries contained a greater presence of *Wolbachia* sp. wAlbB, *Wolbachia* sp. wAlbA, and HALO1.

### 3.3 Mosquito microbiome is partially nested within the environment

**Figure 2:**
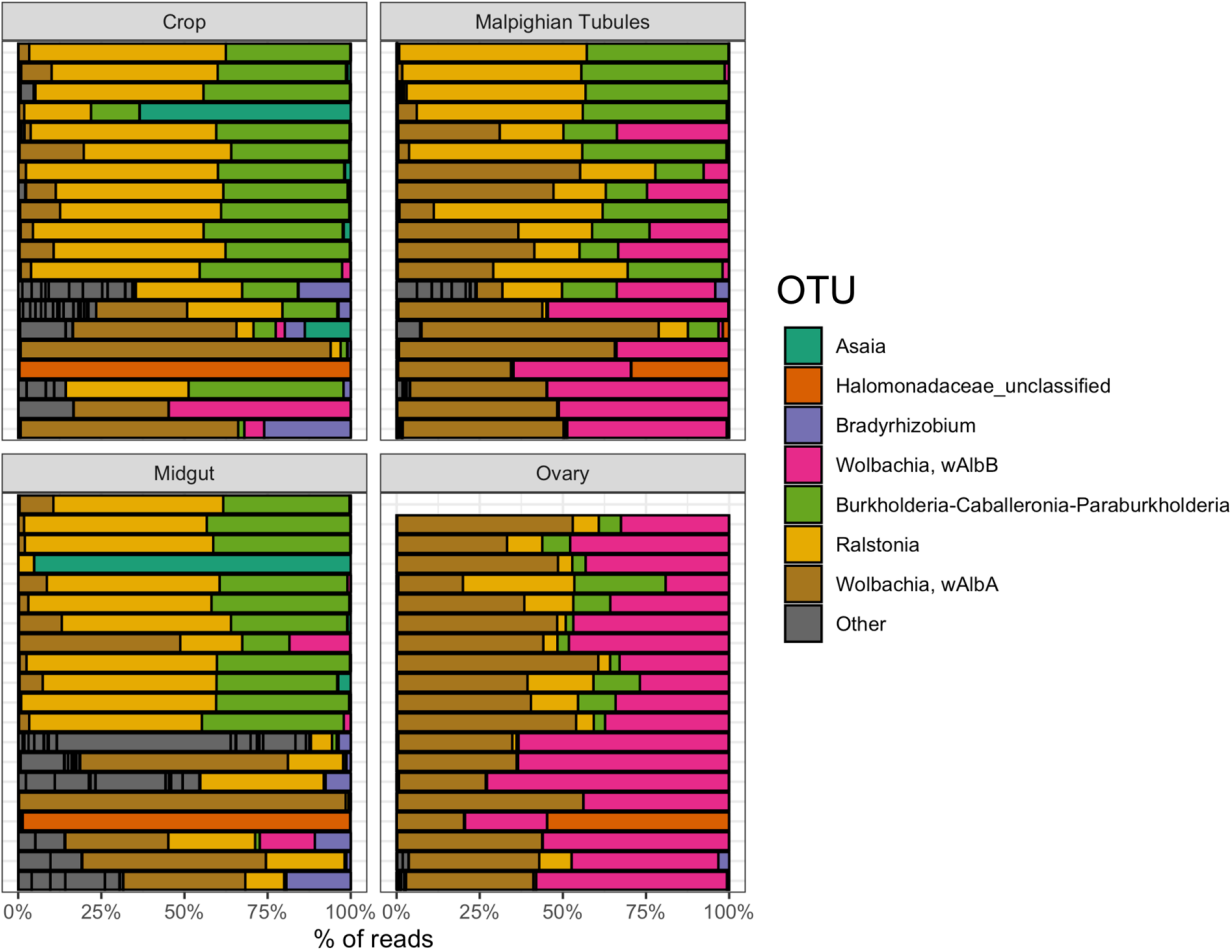
Stacked bar plot showing the prevalence of bacterial taxa within mosquito samples (crop, midgut, Malpighian tubules, and ovaries). Cell values are calculated as proportions across rows. Rare taxa with proportions <3500 total reads were denoted as “other.” See Supporting Information: Table S4 for the abundance of taxa across tissue types.

Ecological network analysis indicated that across all sample types, low‐diversity microbiomes were nested subsets of higher diversity microbiomes. Specifically, when environmental samples (soil, plant nectar, and water) were run as individual sample types, the cumulative data set showed a significant pattern of nestedness (nested temperature = 13.6, *p* = 0.001, null expected range = 31.0–32.3 (95% confidence interval, CI); Figure 2a). We wanted to further understand if this pattern was driven by (i) mosquito tissues being nested in environmental samples, (ii) environmental samples being nested in other environmental samples, (iii) or mosquito tissue samples being nested in other mosquito tissue samples. *A. albopictus* tissue microbiomes remained highly nested within a single variable that summed detections among all the environmental sample types, as compared to null expectations (nested temperature = 25, *p* = 0.001, null expected range = 32.2–33.0 (95% CI); Figure 2b). However, when we removed the environmental data, and only compared mosquito tissues, this nestedness pattern was no longer detected (nested temperature = 0.87, *p* = 0.9, null expected range = 34.7–35.1 (95% CI); Figure 2c). This ruled out a hypothesis that mosquito tissues may be nested along the alimentary canal, with a high diversity crop providing subsets for the midgut and Malpighian tubules that occur more posteriorly. Cumulatively, this suggests that the nestedness of all the sample types is primarily driven by mosquito tissues nesting in environmental samples.

**Figure 3:**
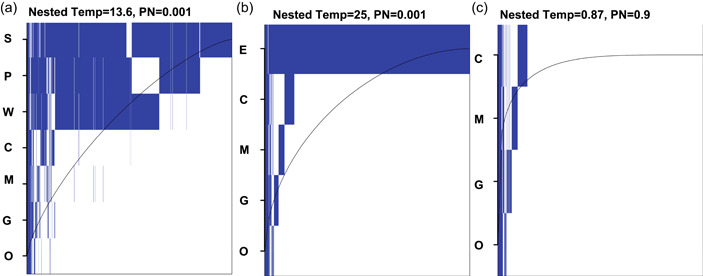
Nestedness plot of bacterial microbiome aggregated by environmental sample and mosquito tissue types. Sample abbreviations: crop (C), midgut (G), Malpighian tubule (M), ovary (O), environment (E), soil (S), plant nectar (P), water (W). (a) Environmental samples are collapsed and compared against individual mosquito tissues (C, G, M, and O). (b) All sample types are compared for nestedness temperature. (c) Only mosquito tissues (c, g, m, and o) are compared for nestedness temperature. Bacterial taxa presence in each sample type is represented as a blue rectangle. Bacterial taxa are organized by residence (left to right) across the different sample types, and rows are ordered from the highest species richness to the lowest species richness (top to bottom). The curved line represents the “Fill line.” If all bacterial taxa occurred above the “Fill line,” then the system would be considered to be perfectly nested (PN).

**Figure 4:**
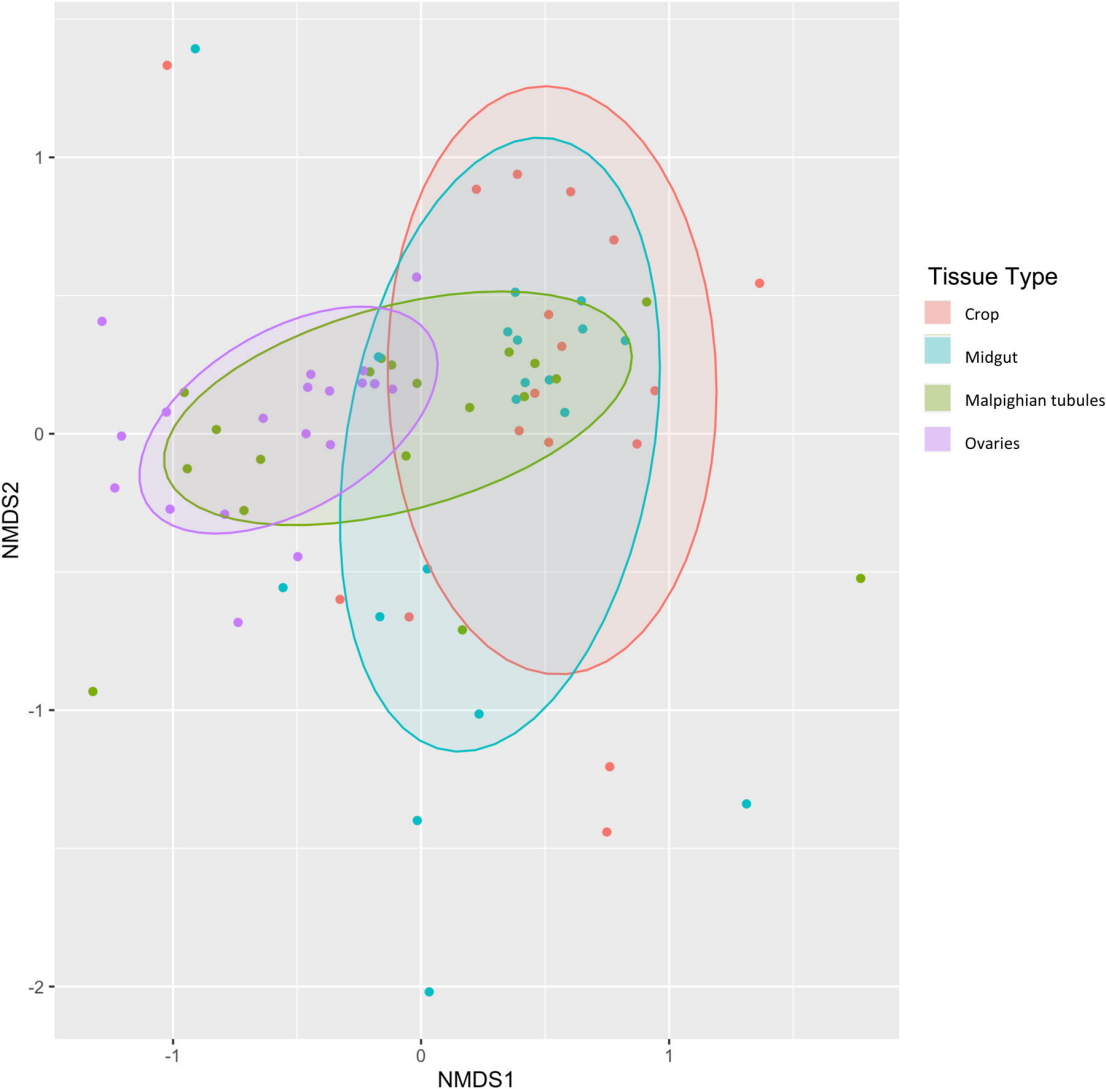
Nonmetric multidimensional scaling (NMDS) demonstrating the composition of bacterial symbionts that assembled within distinct mosquito tissues (crop, midgut, Malpighian tubules, and ovaries) and their respective modules. Distances are based on Bray–Curtis dissimilarity. The stress value of the NMDS is 0.193.

While mosquito tissues derive part of their microbiome from environmental sources, specialization network analysis (*H*2′ = 0.19, *p* = 0.001) indicated that the bacterial symbionts within the mosquito corpus are specialized in specific tissues compared to null expectations. Network modularity assesses link‐rich clusters in a community and allows the identification of species assemblages across ecological communities. The analysis indicated that mosquito tissues were modular (*Q* = 0.26, *z* = 1449, *p* = 0.0001) as compared to null expectations (Figure 3). Two modules were identified from this analysis, crop + midgut and Malpighian tubule + ovary. Further corroborating this finding, GLMMs that tested how tissue‐specific mosquito microbiomes partitioned among hypothetical groupings of tissues indicated that the models that incorporated the groupings (cg‐mo and c‐g‐mo) best fit the data (Table 4). Respectively, models that also included different combinations of hypothetical groupings fit the data poorly (Supporting Information: Table S6). The model that incorporated a cg‐mo grouping variable (AICc weight = 0.575) was 1.7 times more likely than the model that assumed a c‐g‐mo grouping variable (AICc weight = 0.34), though both had a delta AICc < 2.0. One module was shared between the crop and midgut tissues and included several symbionts such as *Wolbachia* sp. wAlbA, *Wolbachia* sp. wAlbB, *Ralstonia* sp., *Burkholderia* sp., HALO (an unclassified Halomonadaceae lineage), and *Asaia* sp. (Supporting Information: Table S6). A second module was shared between the Malpighian tubule and ovary tissues and included the symbionts *Wolbachia* sp. wAlbA, *Wolbachia* sp. wAlbB, and a *Burkholderia* sp., ERWIN1, HALO1, *Xanthobacter* sp., and ENTERO1. An NMDS analysis visually corroborated the clustering pattern of crop–midgut and Malpighian tubule–ovary tissues (Figure 3) and demonstrated distinct groups of bacterial symbionts that clustered according to tissue types (stress level = 0.193). While community composition among tissue types varied (Table 4), the NMDS ordination and the GLMM analysis that grouped different combinations of mosquito tissues suggest a more similar microbiome composition within the crop and midgut, and Malpighian tubule and ovaries, respectively (Figure 4).

**Table 4:** Generalized linear mixed model demonstrating ΔAICc values of distinct models that partitioned variance in the composition of the microbiome among different hypothetical groupings of tissues.

Fixed effects	Random effect	ΔAICc	*df*	AICc weight	Hypothesis
cg‐mo	ASV, ASV × cg‐mo	0	7	0.575	Mosquito microbiome partitions among two modules: crop + midgut and Malpighian tubules + ovaries.
c‐g‐mo	ASV, ASV × c‐g‐mo	1	8	0.34	Mosquito microbiome partitions among three modules: crop; midgut; Malpighian tubules + ovaries.
m‐o‐cg	ASV, ASV × m‐o‐cg	4.6	8	0.058	Mosquito microbiome partitions among three modules: Malpighian tubules; ovaries; crop + midgut.
c‐g‐m‐o	ASV, ASV × c‐g‐m‐o	6.2	9	0.026	Mosquito microbiome partitions among four modules: crop; midgut; Malpighian tubules; ovaries.
Null model	ASV	141.8	5	<0.001	

*Note*: Modules are separated by a hyphen (e.g., cg‐mo indicates the crop and midgut as a module, and the Malpighian tubule and ovary as a separate module; Table 1).

Abbreviations: AICc, corrected Akaike information criterion; ASV, amplicon sequence variant; c, crop; g, midgut; m, Malpighian tubule; o, ovary.

## 4 DISCUSSION

The assembly of metazoan‐associated microbiomes is a dynamic process that leads to substantial diversity and variation between individuals both within and among host populations. One of the pressing challenges of microbiome science is understanding the drivers of this variation (Falony et al., 2016; McLoughlin et al., 2016; Miller et al., 2018). Mosquitoes represent a particularly suitable system to explore the drivers of this variation, due to substantial changes in microbiome composition among individual mosquitoes. In this study, we use various ecological network analyses to clarify how the microbiome diversity of the mosquito compares to that of environmental substrates that mosquitoes interact with over their complex life cycle. Additionally, we clarify how this diversity partitions within mosquito host tissues. Our observed patterns suggest that most of the mosquito microbiome is sourced and selected from the greater diversity of microbes within the environment, and that microbial taxa differentially colonize different tissues within the mosquito (Figure 4).

The diversity of the *A. albopictus* microbiome represents a nested subset of the diversity of microbes in the mosquito environment. In biogeography, patterns of nested biodiversity of distinct ecological communities are indicative of a source–sink relationship in species diversity (Wright et al., 1997), and these dynamics manifest as low‐diversity communities nested in high‐diversity communities. For instance, the fundamental theory of island biogeography assumes that low‐diversity species assemblages on islands form via dispersal from high‐diversity species assemblages on the mainland (MacArthur & Wilson, 2001; Wright et al., 1997). Our data support a consistent assumption in the literature that has been inadequately verified by real data, namely, that mosquito microbiomes (and potentially other metazoans) obtain most of their microbial symbiont diversity from their microbial‐rich environment. Indeed, our 16S‐based data suggest that the adult female mosquito microbiomes were almost entirely composed of microbes that were found in three important environmental substrates that mosquitoes interact with over their life cycle, including water, soil, and plant nectar. While this work implies a source–sink relationship shaping patterns of microbial diversity, future studies should employ more precise molecular markers and experimental techniques that might delimit strain‐level variation shared between microbes in environmental substrates or host‐associated microbiomes. We have demonstrated that mosquitoes can source symbionts from the microbiomes of environmental substrates. This has important implications for mosquito microbiome assembly and the consequences for mosquito health and disease vectoring capability (Seabourn et al., 2020).

While we find that the cumulative diversity of the mosquito microbiome is largely shared with their environment, we also demonstrate that this diversity partitions differentially among distinct tissues in the mosquito corpus. While the associated microbiomes of the crop, midgut, Malpighian tubules, and ovaries were subsets of the environmental microbiome, they did not nest significantly within themselves (i.e., low diversity tissue microbiomes were not subsets of higher diversity tissue microbiomes within a host mosquito). Instead, microbial symbionts were significantly specialized among mosquito tissues and organized into two modules of commonly co‐occurring bacterial symbionts: those in the digestive tissues of the crop and midgut and those in the *Wolbachia*‐harboring tissues of the Malpighian tubules and the ovaries.

Multiple hypotheses might explain the apparent tissue specialization and modularity identified in this study. There is growing evidence that hosts actively maintain symbiosis that will support host organismal performance (Bascuñán et al., 2018; Grond et al., 2020). Symbionts provide essential functions to their host (Martin et al., 2019), and selection may favor hosts that differentially associate with symbionts and harbor them in the right tissues to provide these functions (Mandel & Dunn, 2016). Interestingly, these data suggest a module between the microbiomes of the crop and midgut tissues, two tissues that have important functions associated with food digestion and nutrition (Calkins et al., 2017; Guégan et al., 2020). Given the shared physiological processes of the crop and midgut (i.e., nutritional functions) and their close proximity (i.e., connection via the alimentary canal), it is plausible that the host would select for symbionts capable of supporting this broad physiological function and that connectivity via the alimentary canal permits for frequent dispersal between these tissue types; however, the mechanism for this selection and indeed the function of many of the symbionts such as *Burkholderia* sp. would need to be studied further. The symbiont *Burkholderia* sp., Acetobacteraceae found within the crop and midgut tissues may assist with bloodmeal digestion by detoxifying ammonia in the gut (Feldhaar et al., 2007; Villegas et al., 2023).

Module formation may also recapitulate developmental processes. For example, Malpighian tubules are excretory tissues responsible for osmotic balance and excretion of waste. Unlike most larval stage tissues, Malpighian tubules remain intact during metamorphosis in *Drosophila* (Chavshin et al., 2015; Faria & Sucena, 2013), while the ovaries and testes develop from mesodermal follicle precursor cells (female‐only), genital disc, and pole cells (male and female). Recent studies have highlighted the possibility that Malpighian tubules facilitate the transstadial transmission of bacterial symbionts from the larval to the adult stage (Chavshin et al., 2015). In this study, the Malpighian tubules have the second greatest species diversity, higher than the microbiomes of the midgut and ovaries (Table 1) and may serve as a source for transstadial transmission of environmentally acquired bacterial symbionts to redeveloped tissues following metamorphosis. For instance, the Malpighian tubules served as a refugium for the bacterial symbiont *Pseudomonas*, facilitating its transmission from the larval stage to the adult stage in *Anopheles stephensi* mosquitoes (Chavshin et al., 2015). Previous studies have also demonstrated that Malpighian tubules harbor large numbers of *Wolbachia* sp. bacteria and may persist through vertical transmission (Faria & Sucena, 2013)

While the environment within a host plays an important role in microbiome assembly and maintenance, microbial biology also influences microbial growth rates, which must contribute to shaping their distribution among mosquito tissues. For instance, NITRO1 (an unclassified Nitrososphaeraceae lineage), which is known to contain ammonia oxidizing strain, was entirely restricted to the crop, an area exposed to ammonia‐rich substrates due to its proximity to the environment. Other studies have also found tight associations between *Aedes* crop tissues and Acetobacteraceae (Feldhaar et al., 2007). Furthermore, the ovaries were the only site for bacterial symbionts *Pseudomonas*, *Acinetobacter*, Methyloligellaceae (METHYL1), *Sphingomonas*, and *Methylobacterium*. Overall, these factors determine not only microbial survival but also microbe colonization potential that may be dependent on tissue structure, physiological environment, neighboring microbes, and the intramicrobial dynamics of mosquito microbiotas.

Broadly, these data demonstrate that mosquito tissue microbiomes are nested within environmental sources (soil, plant nectar, and water), indicating that mosquito microbiotas may be sourced from specific environmental sources like plant nectar, soil, and aquatic habitat (i.e., water) (Boet et al., 2020). Specifically, 98% of *A. albopictus* microbiome diversity is shared with environmental sources. Additionally, while mosquito microbial symbionts derive from environmental sources, they also assemble into distinct microbiomes across tissue types (crop, midgut, Malpighian tubule, and ovaries) and form specialized modules based on commonly co‐occurring symbiont species across tissue groups (specifically, the digestive tissues of the crop and midgut and the *Wolbachia*‐harboring Malpighian tubules and ovaries). While this study cannot differentiate the mechanisms that shape this selective assembly of mosquito symbionts from the environment, a plausible scenario would involve varied dispersal and filtering of symbionts from the environment, niche effects within the mosquito that sort species based on growth optima, and physiological mechanisms that allow hosts to select microbes and alter growth rates. Future studies should seek to clarify the specific mechanisms that govern microbiome assembly within mosquito hosts. Further understanding of the mechanisms driving module formation, niche‐driven assembly, and host‐mediated microbe selection demonstrated in this study will shed light on the complexity of ecological networks and can enrich our understanding of microbiome assembly within hosts. Additionally, clarifying drivers of microbiome variation in specific mosquito tissues has substantial implications for improving disease‐mitigating strategies (Gao et al., 2021; Wilke & Marrelli, 2015).

## AUTHOR CONTRIBUTIONS

**Priscilla S. Seabourn**: Conceptualization (equal); data curation (equal); formal analysis (equal); funding acquisition (equal); investigation (equal); methodology (equal); project administration (equal); resources (equal); software (equal); supervision (equal); validation (equal); visualization (equal); writing—original draft (equal); writing—review and editing (equal). **Danya E. Weber**: Investigation (equal); resources (equal); writing—review and editing (equal). **Helen Spafford**: Conceptualization (equal); project administration (equal); visualization (equal); writing—review and editing (equal). **Matthew C. I. Medeiros**: Conceptualization (equal); data curation (equal); formal analysis (equal); funding acquisition (equal); investigation (equal); methodology (equal); project administration (equal); resources (equal); software (equal); supervision (equal); writing—review and editing (equal).

## CONFLICT OF INTEREST STATEMENT

None declared.

## ETHICS STATEMENT

None required.

## Supporting information

Supporting information.

## Data Availability

All relevant data are included within the article and its Supporting Information file. Raw sequence reads are deposited in Dryad: https://doi.org/10.5061/dryad.3xsj3txm7.

## 1

Figure A1
